# Active permanent greening – a new slope greening technology based on mineral solubilizing microorganisms

**DOI:** 10.3389/fpls.2023.1219139

**Published:** 2023-08-29

**Authors:** Lingjian Wang, Xinggang Tang, Xin Liu, Jinchi Zhang

**Affiliations:** ^1^ Jiangsu Province Key Laboratory of Soil and Water Conservation and Ecological Restoration, Nanjing Forestry University, Nanjing, Jiangsu, China; ^2^ Jiangxi Institute of Land Space Survey and Planning, Nanchang, Jiangxi, China; ^3^ Technology Innovation Center for Land Spatial Eco-protection and Restoration in Great Lakes Basin, MNR, Nanchang, Jiangxi, China

**Keywords:** slope management, mineral weathering, greening, vegetation restoration, analytic hierarchy process

## Abstract

**Introduction:**

With social and economic development and the associated large-scale exploitation of natural resources, the number of slopes has significantly increased. As slope instability can lead to serious geological disasters, the ecological protection and reconstruction of slopes has become a hot topic of common global concern.

**Methods:**

In order to achieve scientific slope management and overcome the difficulty of maintaining slope greening in the long term, this study explored eight strategies (A, B, C, AB, AC, BC, ABC, CK), involving different patented mineral solubilizing microorganisms (MSMs), and analyzed the field application of active permanent greening (APG) based on MSMs.

**Results:**

The results revealed that MSMs significantly increased the content of effective metal ions and available nutrients in soil and enhanced soil enzyme activity. Among all strategies, strategy A showed significant superiority, with soil effective calcium, magnesium, potassium, nitrogen, phosphorus and organic matter contents increasing by 51.62%, 55.41%, 30.42%, 39.77%, 181.69% and 76.92%, respectively, while urease, sucrase and peroxidase activities increased by 89.59%, 74.68% and 85.30%. MSMs strongly promoted the growth of Amorpha. Strategy A showed the best performance, with plant seedling height, ground diameter, leaf area, root length, and root volume increasing by 95.75%, 47.78%, 124.14%, 108.83%, and 139. 86%, respectively. According to a comprehensive evaluation using the entropy-analysis hierarchy process, strategy A has great potential for application. The field test results verified that APG has significantly better greening performance than the traditional greening method, with high vegetation cover and stable soil layer.

**Discussion:**

The results of this study provide a reliable practical basis and technical reference for the development, promotion, and application of APG.

## 1 Introduction

With the development of science and technology, human ability to exploit nature has been increasing, and the development of mineral resources and large-scale engineering constructions, such as paving roads and bridges, have provided a guarantee for human production and life while destroying the natural ecological balance (Zhai et al., 2021; Li et al., 2022a). Furthermore, the number of exposed slopes have significantly increased (Ahirwal and Maiti, 2021). Exposed slopes can cause a series of environmental problems, such as soil erosion, landslides, mudslides and local microclimate deterioration, seriously threatening the safety of human life and property (Stern et al., 1996; Zhu et al., 2021). Moreover, the ecological restoration of exposed slopes through natural means is difficult and time-consuming (Shen et al., 2023). Therefore, many researchers have devoted themselves to find scientific, effective, and economical methods for managing slopes and improving slope greening.

The concept of ecological protection had an early start in developed countries. Japan carried out the barren mountain treatment project as early as 1633, developed a more practical spray seeding greening technology in 1958, and gradually developed several methods from the 70s to 90s, such as the fiber soil greening method, high sub-glomerate spraying technology (SF greening method), continuous fiber greening method (TG greening method), which were promoted to China, the United States, etc. In recent years, Japan began to fully implement a multi-layer three-dimensional greening system (Geng et al., 2013; Peng et al., 2015; Xiao et al., 2015; Li et al., 2019). In European countries, ecological slope protection is being used primarily for the stability of embankments and traffic line slopes, and related research mostly focus on slope protection under rainwater erosion (Melillo et al., 2015; Fusco et al., 2019). Since the 1930s, after several ecological disasters in the United States, importance has gradually been given to slope ecological protection, relevant technologies have been developed, research on greening substrates has been gradually advanced, and mechanized construction has been fully realized (Tyser et al., 1998; Paschke et al., 2001; Zelnik et al., 2008). Compared with western developed countries, research on slope greening technology in China began much later, starting in the 1980s. After a series of research exploration and engineering practices, it has been vigorously developed and technologies such as three-dimensional vegetation network, hydroseeding grass planting, thick matrix spraying, hybrid spraying vegetation, and grass concrete planting have been gradually formulated (Zhao et al., 2018; Ma et al., 2020; Yan et al., 2020; Li et al., 2022b). However, existing technologies still have many issues to be resolved, such as nutrient loss from the cover soil, the inability of plant roots to penetrate deep layers, and the threat of dry heat to vegetation. Consequently, it is difficult to maintain the greening effect in the long term.

Soil microorganisms play an important role in biogeochemical cycles (Bertrand et al., 2015). Microorganisms can provide nutrients to plants by solubilizing bound mineral components through acidolysis, complexation, chelation and exchange reactions (Vaid et al., 2014; Kamran et al., 2017; Berde et al., 2021). A number of strains of the genera *Bacillus* and *Streptomyces* have been found to be capable of releasing metal ions from minerals (Sindhu et al., 2014; Cumpa-Velásquez, 2021). In addition, microorganisms can directly or indirectly regulate nutrient cycling in soil-plant ecosystems, change soil fertility and structure, promote plant growth, suppress plant diseases, and improve plant resistance (Kour et al., 2019; Sattar et al., 2019) through biological nitrogen fixation (Wu et al., 2019), phytohormone production (Kim et al., 2017; Zhao et al., 2022), iron carrier regulation (Kumar et al., 2019), and secondary metabolite reactions (Franzluebbers, 2002). Approximately 60 000 strains of Bacillus thuringiensis have been preserved worldwide which are widely used in plant protection and pest control (Akhtar et al., 2021; Isayama et al., 2021). Considering the importance of microorganisms in the plant-soil ecosystem, effective soil microorganisms can be combined with traditional spraying technology to utilize the role of microorganisms in accelerating the weathering of rocks, improving the soil nutrient environment, and promoting the growth of plants and roots, thus fundamentally overcoming the defects of traditional spraying technology in which plant roots cannot penetrate deeply and the greening effect is difficult to maintain. This provides a new way of thinking for the improvement and updating of slope greening technology.

Previously, we isolated a variety of microorganisms from the weathered rock wall soil of Nanjing Mufu Mountain and selected 16 of them for culture tests and investigating solubilization mechanisms. Four typical strains were selected for patent protection based on the test results. We found that these strains positively affected the release of mineral metal ions, plant and root growth, and photosynthesis of plants (Wu et al., 2017a; Wu et al., 2017b; Li et al., 2020; Jia et al., 2021; Wang et al., 2022). However, the comprehensive effects of these factors on soil, plants, and roots have not been considered yet, nor have they been integrated with revegetation construction techniques. On the whole, they have not really been applied in slope management practice.

In order to comprehensively analyze the effects of different mineral-solubilizing microorganisms (MSMs) on soil-vegetation ecosystems, we conducted a series of controlled experiments to rank the effects of different strategies and initially applied the active permanent greening (APG) method based on MSMs. The objectives of this study were as follows: (1) to study the effects of MSMs on soil, plant, and root systems; (2) to comprehensively analyze and evaluate the application effects of strategies involving different MSMs; and (3) to evaluate the practical application of the field simulation experiments by the APG method. The results of this study will enrich existing information on the effects of MSMs on soil, plant, and root systems and guide further practical application efforts. More importantly, this study combines soil effective microorganisms with traditional engineering greening techniques, providing feasible directions and strategies for the improvement and innovation of greening techniques for slope revegetation, and providing practical basis and technical support for the application and promotion of the APG method.

## 2 Materials and methods

### 2.1 Microorganism strains

The bacterial strain *Bacillus thuringiensis* NL-11, the fungal strain *Gongronella butleri* NL-15, and the actinomycete strain *Streptomyces thermocarboxydus* NL-1, isolated and screened from the surface of weathered rock walls of Mufu mountain (rock properties as shown in **Table 1**), were obtained from the Soil and Water Conservation Laboratory, Department of Forestry, Nanjing Forestry University (Jiangsu, China). These strains have been conserved in the China Typical Culture Conservation Center (CCTCCNO: M2012453, CCTCCNO: M2012454, and CCTCCNO: M2012460, respectively) (Guanglin W. et al., 2014; Jinchi et al., 2014a; Jinchi et al., 2014b). The well-preserved strains were activated using Nutrient Agar (Peptone, 10.0 g/L; Beef Extract Powder, 3.0 g/L; NaCl, 5.0 g/L; Agar,15.0g/L), Potato Sucrose (Potato infusion powder, 7.0 g/L; Sucrose, 20.0 g/L; Agar, 20.0 g/L) and Actinomycetes Culture (Soluble Starch, 20.0 g/L; NaCl, 0.5 g/L; KNO_3_, 1.0 g/L; KH_2_PO_4_·3H_2_O, 0.5 g/L; MgSO_4_· 7H_2_O, 0.5 g/L; FeSO_4_·7H_2_O, 0.01 g/L; Agar, 15.0 g/L) medium. To achieve appropriate survival numbers, cultures of the strains were prepared by inoculating the activated strains individually in Nutrient Broth (Peptone, 10.0 g/L; Beef Extract Powder, 3.0 g/L; NaCl, 5.0 g/L), Potato Liquid (Potato dip powder, 6.0 g/L; Glucose, 20.0 g/L; Chloramphenicol, 0.1 g/L) and Actinomyces liquid (Soluble Starch, 20.0 g/L; NaCl, 0.5 g/L; KNO_3_, 1.0 g/L; KH_2_PO_4_·3H_2_O, 0.5 g/L; MgSO_4_· 7H_2_O, 0.5 g/L; FeSO_4_·7H_2_O, 0.01 g/L) medium.

**Table 1:** Elemental composition of minerals.

Element	CaO	MgO	K_2_O	Fe_2_O_3_	Al_2_O_3_	SiO_2_	Na_2_O	Others
**Composition(W/%)**	62.34	27.93	1.75	3.00	0.61	1.35	0.04	2.95

### 2.2 Plants material and soil strategies

Amorpha (*Amorpha fruticosa* Linn.) was selected as a salt and drought tolerant engineering green species for use in this study, and seeds were provided by Shun Hua Ge Flower Co. A pot experiment was conducted using the strain culture mixed thoroughly with an appropriate amount of sterilized soil (Soil properties were: effective nitrogen content of 97.75 mg·kg^-1^; effective phosphorus content of 5.97 mg·kg^-1^; effective potassium content of 115.40 mg·kg^-1^; organic matter content of 11.8 g·kg^-1^). For the control group, sterile water was used. Eight strategies were set up, with three replicates for each strategy. The strain configurations for the different strategies are shown in **Table 2**. In each strategy, the initial moisture content was set at 0.3 m^3^/m^3^ (V/V). After three months of routine custodial culture, soil, plant, and root samples were collected from the pots for measurement.

**Table 2:** Strategies for potting experiments.

Strategies	Strain configuration	Strategies	Strain configuration
**A**	NL-11	**AC**	NL-11 ×NL-1
**B**	NL-15	**BC**	NL-15 ×NL-1
**C**	NL-1	**ABC**	NL-11×NL-15×NL-1
**AB**	NL-11×NL-15	**CK**	No added MSMs

### 2.3 Variable selection and measurement methods

#### 2.3.1 Variable selection

Sixty appropriate variables were collected from studies in the fields of soil microbiology and slope vegetation restoration to prepare a questionnaire. Twenty- three main variables were extracted from the expert survey results, grouped into three main categories (soil, plant, and root system), and tested in controlled experiments. The experts involved in the survey had extensive experience in soil microbial applications and slope revegetation, with one or more published reports on the research topic or experience leading practical work on revegetation projects.

#### 2.3.2 Determination of soil properties

Soil effective calcium, magnesium and potassium ion concentrations were measured through atomic absorption spectrophotometry (AAS) (Perkin Elmer SIMMA 6000, Norwalk, USA) (Behera et al., 2021). The concentration of AN was analyzed using the NaOH hydrolysis diffusion method. Available phosphorus was extracted using sodium bicarbonate and then measured by the molybdenum-blue method(Liu et al., 2022). Soil urease activity was determined by incubating 10 g of soil with 10 ml of 10% urea solution for 24 h at 37°C. Ammonium released from urea hydrolysis was quantified in a UVS at 578 nm (Akhtar et al., 2018). Soil catalase (CAT) activity was measured by incubating 2.0 g of soil, 40 ml of distilled water, and 5 ml of 0.3% H_2_O_2_ in a mixture (shaking at 150 rpm for 20 min), which was titrated with 0.1 mol L^-1^ KMnO_4_ and the volume of each titration was recorded. Sucrase activity was determined using sucrose as the soil, and the activity was expressed as the mass of glucose per gram of soil after 24 h (Ren et al., 2018).

#### 2.3.3 Determination of plant properties

Height, diameter and leaf area of plants were determined using a measuring tape, vernier caliper and LI-3000C portable area metepr (Li-Cor Inc., USA), respectively. For the extraction of chlorophyll pigment, plant samples (0.5 g) were dipped in 85% acetone kept in the dark. The supernatant was collected and centrifuged at 600 rpm for 15 min and absorbance was calculated at 645 and 663 nm. Total chlorophyll (Chl *a* + Chl *b*) was measured as the sum of chlorophyll a (Chl *a*) and chlorophyll b (Chl *b*) (Kumar et al., 2021). The total soluble sugar content of the plants was determined using the anthranilic sulfuric acid method (Abdel Latef and Tran, 2016). The protein content was analyzed using Coomassie Brilliant Blue G-250 as a dye and albumin as a standard (Habiba et al., 2015).

#### 2.3.4 Determination of root system properties

Root images were analyzed using WinRhizo software (Regent Instruments Canada Inc) to derive root length, root surface area, root volume, and root projected area. Total root length and total root surface area were calculated for each strategy. Root system vigor was measured using the triphenyltetrazolium chloride (TTC) method described by Chen et al. (Chen et al., 2018).

### 2.4 Data analysis

**Figure 1:**
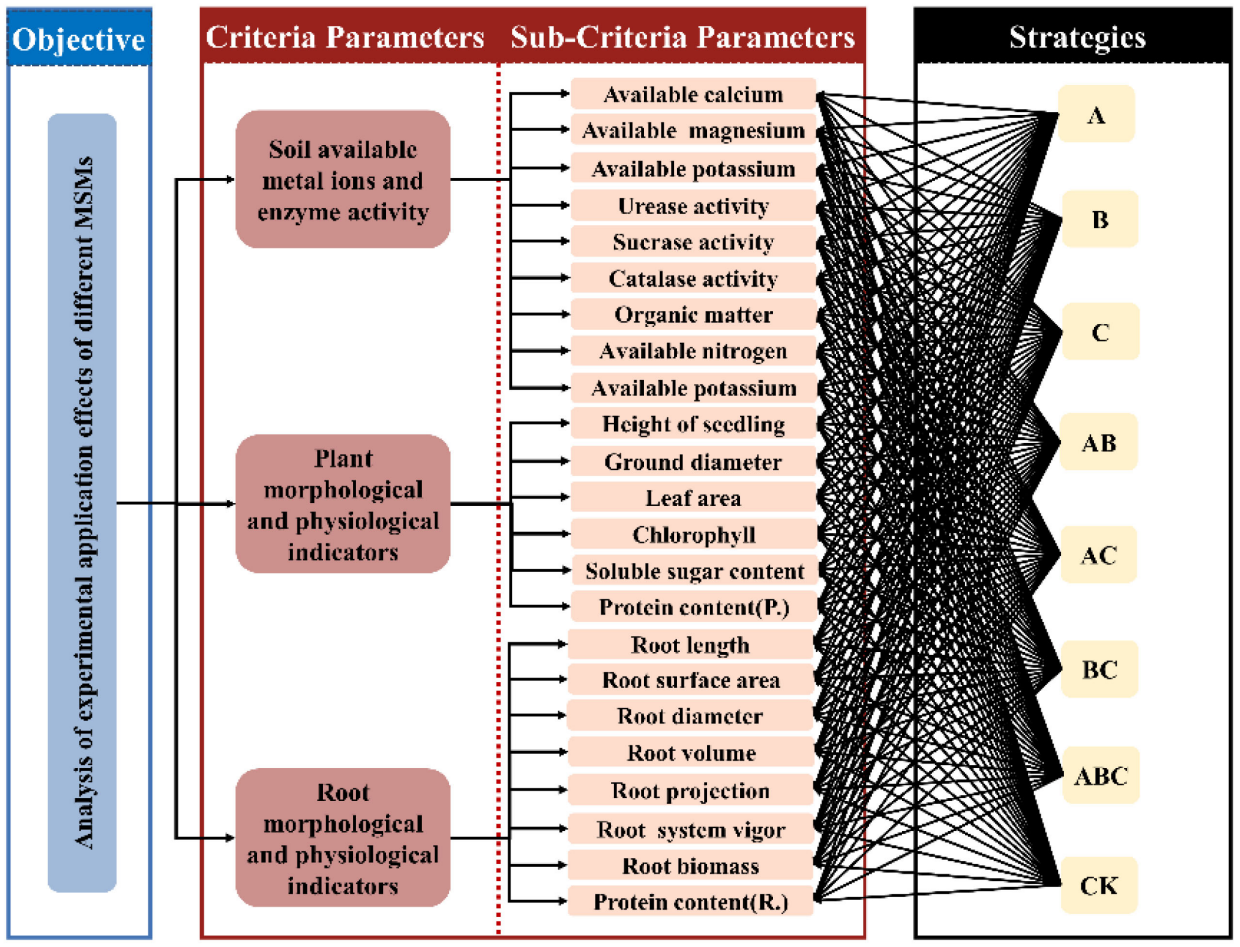
Schematic diagram of the hierarchy of different strategies.

The analytic hierarchy process (AHP) method is a decision-making method that decomposes elements related to decision making into levels of objectives, criteria and options, and analyzes and models complex systems qualitatively and quantitatively. The entropy method is a mathematical method used to determine the degree of dispersion of a given indicator. The larger the degree of dispersion, the stronger the influence of that indicator on the comprehensive evaluation. Therefore, the weight of each indicator can be calculated on the basis of the degree of dispersion of each indicator. The hierarchical structure of the analysis process for this study is shown in **Figure 1**. The weights of the data for each category were calculated based on the expert survey results using AHP, and the weights of each indicator data were calculated based on the results of controlled experimental tests using the entropy method. The integrated assignment method was used to fuse the weights calculated based on AHP and the entropy method to compare and calculate each indicator between each level and determine the importance of different schemes. Analysis of variance (ANOVA) was performed on all strategies for soil characteristics, plant characteristics, and root characteristics using SPSS 26.0 software, and the means were compared using Duncan’s test. Values of P < 0.05 were considered to indicate significant differences.

## 3 Results

### 3.1 Effect on soil

**Figure 2:**
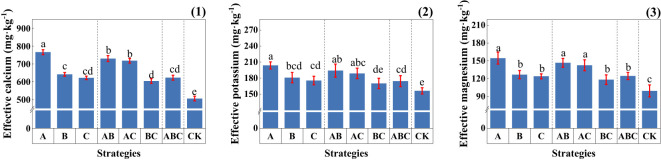
Soil effective metal ion content under different strategies. (1) Soil effective calcium content (mg·kg^-1^); (2) Soil effective potassium content (mg·kg^-1^); (3) Soil effective magnesium content (mg·kg^-1^);Measurements were taken from three soil samples of three potted replicates per strategy. Each value is the mean value ± standard error (SE) of three independent replicates. Different lowercase letters represent significant differences (P ± 0.05) according to the Duncan test (ANOVA).

After the addition of MSMs to the soil, the effective calcium, magnesium, and potassium contents of the soil significantly increased, as shown in **Figure 2**. The most significant promotion effect was observed under strategy A.

**Figure 3:**
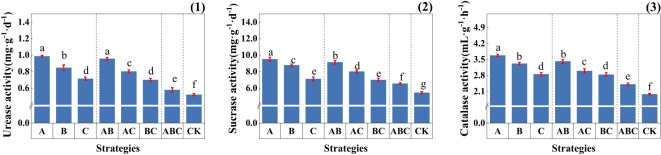
Soil enzyme activity under different strategies. (1) Soil urease activity (mg·g^-1^·d^-1^); (2) Soil sucrase activity (mg·g^-1^·d^-1^); (3) Soil catalase activity (mL·g^-1^·h^-1^); Measurements were taken from three soil samples of three potted replicates per strategy. Each value is the mean value ± standard error (SE) of three independent replicates. Different lowercase letters represent significant differences (P < 0.05) according to the Duncan test (ANOVA).

As shown in **Figure 3**, the addition of MSMs significantly enhanced the enzyme activity of the soil. It is noteworthy that the most prominent increases in the effective metal ion content and enzyme activity in soil were observed under strategies A, AB, and AC, which included NL-11.

As shown in **Table 3**, the addition of MSMs obviously improved the nutrient content of the soil, among which strategy A showed superior performance, with the content of soil organic matter, available nitrogen, and available phosphorus increasing by 76.92%, 39.77%, and 181.69%, respectively, compared with the control.

**Table 3:** Soil nutrient content under different strategies.

Strategies	Organic matter (g·kg^-1^)	Available nitrogen (mg·kg^-1^)	Available phosphorus (mg·kg^-1^)
**A**	17.94 ± 0.36 a	129.66 ± 4.70 a	17.38 ± 0.29 a
**B**	16.28 ± 0.36 bc	122.22 ± 7.66 ab	13.00 ± 0.35 c
**C**	14.86 ± 0.31 d	117.11 ± 5.95 ab	11.23 ± 0.52 d
**AB**	16.61 ± 0.33 b	126.89 ± 5.48 a	14.54 ± 0.48 b
**AC**	14.29 ± 0.24 e	111.99 ± 3.91 abc	12.79 ± 0.39 c
**BC**	15.98 ± 0.31 c	115.53 ± 3.93 ab	10.68 ± 0.39 d
**ABC**	11.29 ± 0.21 f	104.92 ± 4.43 bc	9.86 ± 0.41 e
**CK**	10.14 ± 0.16 g	92.77 ± 2.48 c	6.17 ± 0.32 f

Each value is the mean ± standard error of three independent replicates. Different letters represent significant differences (P < 0.05) according to the Duncan test (ANOVA).

### 3.2 Effect on plants growth

The addition of MSMs significantly promoted plant growth, and it is noteworthy that seedling height, ground diameter, and leaf area increased by 95.75%, 47.78%, and 124.14%, respectively, under strategy A, compared with the control (**Table 4**).

**Table 4:** Growth of Amorpha under different strategies.

Strategies	Height (cm)	Diameter (mm)	Leaf area (cm^2^)
**A**	30.87 ± 1.45 a	2.66 ± 0.05 a	214.70 ± 4.59 a
**B**	29.67 ± 0.71a	2.40 ± 0.06 bc	188.25 ± 4.31 c
**C**	26.97 ± 0.90 b	1.94 ± 0.04 e	157.03 ± 2.67 d
**AB**	27.13 ± 0.67 b	2.43 ± 0.04 b	151.57 ± 4.69 b
**AC**	25.10 ± 0.78 c	2.34 ± 0.05 c	139.83 ± 3.30 e
**BC**	21.87 ± 0.91 d	2.14 ± 0.04 d	129.78 ± 3.38 f
**ACB**	19.10 ± 0.96 e	1.81 ± 0.05 f	113.97 ± 3.26 g
**CK**	15.77 ± 0.68 f	1.80 ± 0.04 f	95.79 ± 1.03 h

Each value is the mean ± standard error of three independent replicates. Different letters represent significant differences (P < 0.05) according to the Duncan test (ANOVA).

**Figure 4:**
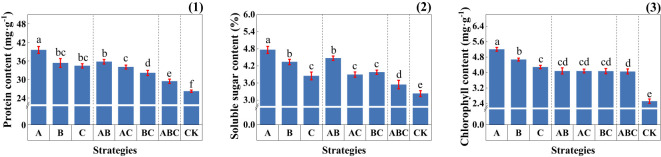
Physiological indicators of plants under different strategies. (1) Root volume (cm^3^) and its grading (%); (2) Root projection area (cm^2^) and its grading (%); (3) Root length (cm) and its grading (%); (4) Root surface area (cm^2^) and its grading (%). Measurements were taken from three plant root samples of three potted replicates per strategy. Each value is the mean value ± standard error (SE) of three independent replicates. Different lowercase letters represent significant differences (P < 0.05) according to the Duncan test (ANOVA).

The addition of MSMs significantly increased the protein and soluble sugar contents of the plants, with strategies A and AB showing higher performance (**Figure 4**). Furthermore, the MSMs had a strong enhancement effect on chlorophyll content Figure, with single strain strategies exhibiting outstanding performance.

### 3.3 Effect on root growth

**Figure 5:**
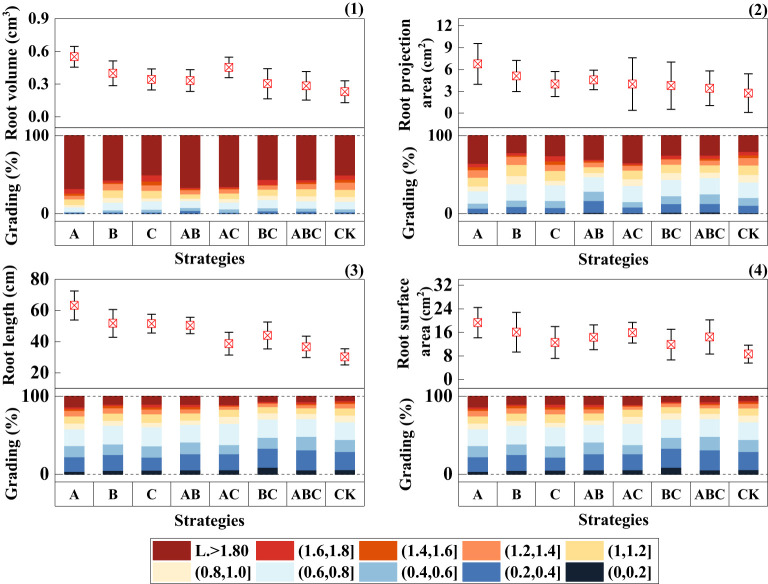
Root growth status under different strategies. (1) Root volume (cm^3^) and its grading (%); (2) Root projection area (cm^2^) and its grading (%); (3) Root length (cm) and its grading (%); (4) Root surface area (cm^2^) and its grading (%). Measurements were taken from three plant root samples of three potted replicates per strategy. Each value is the mean value ± standard error (SE) of three independent replicates.

As shown in **Figure 5**, the addition of MSMs significantly improved root growth, with strategy A showing particularly remarkable performance.

**Figure 6:**
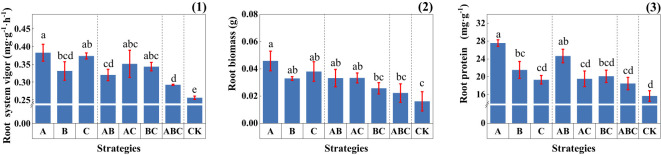
Physiological indicators of root systems under different strategies. (1) Root system vigor (mg·g^-1^·h^-1^); (2) Root biomass (g); (3) Root protein (mg·g^-1^); Measurements were taken from three plant root samples of three potted replicates per strategy. Each value is the mean value ± standard error (SE) of three independent replicates. Different lowercase letters represent significant differences (P < 0.05) according to the Duncan test (ANOVA).

As shown in **Figure 6**, the addition of MSMs enhanced the root vigor of plants. Nevertheless, the four strategies containing NL-15 (B, AB, BC, ABC) showed slightly lower performance.

### 3.4 Comprehensive analysis of different strategies

**Figure 7:**
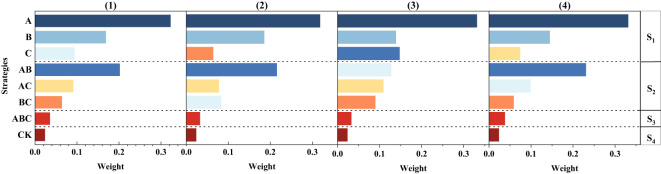
Weight ranking of different strategies. (1) Combined ranking of each strategy based on all variables; (2) Weight ranking of each strategy based on plant class indicators; (3) Weight ranking of each strategy based on root system indicators; (4) Weight ranking of each strategy based on soil indicators. S1: Strategies containing only one type of MSM; S2: Strategies containing two types of MSMs; S3: Strategies containing three types of MSMs of both types; S4: No MSM control group.

Different strategies were ranked using entropy-AHP for the weight analysis of the soil, plant, and root system and the entire system. In terms of the effects of MSMs on plants, strategies A, AB and B were more effective; regarding roots, strategies A, B and C were more effective; regarding soil, strategies A, AB and B were more effective; and regarding all variables, strategies A, AB and B were more effective, as detailed in **Figure 7**. Therefore, strategy A was considered the best strategy in this study.

## 4 Discussion

Slopes are a type of landform that form naturally or through human activities. Unstable slopes are prone to landslides, mudslides, and other disasters, which seriously endanger human life and property. Therefore, slope protection and management technology has been the focus of many scientific and technical efforts. Our research team has been devoted to the investigation of the ecological environment of slopes and the research of management technology for many years. Wu et al. (Wu et al., 2017a; Wu et al., 2017b; Wu et al., 2021; Wu et al., 2022) isolated and screened excellent MSMs according to their influence on mineral weathering, and explored the influence mechanism using genomic and transcriptomic analysis. Jia et al. (Jia et al., 2021) conducted pot experiments and found that MSMs could promote plant growth, and notably, the number of nodules in the roots of plants was significantly elevated. Li et al. (Li et al., 2020; Li et al., 2021a; Li et al., 2021b) investigated changes in plant root characteristics and root reinforcement in soil in response to MSMs and discussed the underlying mechanism. In addition, we also carried out a series of tests and researches on slope spraying substrates, including water retention agents. The results of a large number of studies suggest that the APG method based on MSMs is an effective and feasible method of slope greening, and it has great application value and broad application prospects.

Creation of a fertile substrate is the key to spray seeding technology. Numerous investigations have shown that the weathering of rocks varies considerably in the presence or absence of microorganisms. Mineral-solubilizing microorganisms are able to promote mineral decomposition and weathering through their metabolites, extracellular secretion and redox exchange functions, accelerating the process of rock soilization. (Uroz et al., 2009; Olsson-Francis et al., 2010; Rahimzadeh et al., 2015; Ahmad et al., 2016; Wang et al., 2020) In addition, mineral solubilizing microorganisms can improve soil structure and nutrient conditions (Spaepen and Vanderleyden, 2011; Zhu et al., 2014; Ribeiro et al., 2020), artificially creating soils with high sub-agglomerate structure and inhabiting various soil critters and microorganisms, simulating natural habitats. At present, mineral solubilizing microorganisms have many applications in heavy metal remediation(Mishra et al., 2017; Yin et al., 2019; Fakhar et al., 2020) and microbial metallurgy(Behera and Mulaba-Bafubiandi, 2016; Ilyas et al., 2018; Priya and Hait, 2020), but there is little research in slope engineering management. In this study, MSMs could significantly increase the effective calcium, magnesium, and potassium ion contents of the soil, which is consistent with previous findings (Villarreal Sanchez et al., 2018; Soumare et al., 2022). Strategy A showed the highest performance among the different strategies. It is worth mentioning that both single and two microbial configurations containing bacterial NL-11 (A, AB, AC) showed a good promotion effect on the effective metal ion content of the soil, with AB showing outstanding performance. This indicates that bacterial NL-11 not only possesses a strong mineral solubilization effect, but also shows good adaptability when synergizing with other microorganisms. However, the mixed configuration of the three microorganisms showed mediocre performance, which may be due to the competitive relationship among the three microorganisms, the reason for which needs to be explored in further studies.

Soil enzyme activity plays an important role in soil nutrient availability (Demisie et al., 2014). It is also considered as a potential indicator of soil fertility (Guangming et al., 2017) and a key factor in the functions of forest soil ecosystems. Different soil enzymes have different functions. Soil urease is the only amidase in the soil that can convert urea to useful nitrogen and is closely related to nitrogen in the soil. Soil CAT reduces the damage to plant roots caused by the excessive accumulation of hydrogen peroxide in soil. The results clearly indicate the significant enhancement effect of MSMs on urease and catalase in the soil. Many studies have reported a positive linear relationship between soil urease and catalase activities and total soil N in the presence of microorganisms (Saiya-Cork et al., 2002; Li et al., 2017). Similar findings were found in our previous and current studies. (Jia et al., 2021; Li et al., 2021b). In this study, strategy A with NL-11 was clearly found to have the highest performance among all options. Notably, all two-strain mixed strategies containing NL-11 showed generally satisfactory performances.

Vegetation growth is an important criterion for the comprehensive evaluation of ecological protection and reconstruction of slopes. Microorganisms have been reported to be capable of regulating plant growth and stress resistance through various direct or indirect mechanisms (Pii et al., 2015; Souza et al., 2015). Microorganisms can also contribute to plant growth by directly providing deficient nutrients to plants (Richardson et al., 2009; Di Benedetto et al., 2017) through nitrogen fixation, phosphorus solubilization (Bononi et al., 2020), and metal mobilization (Sindhu et al., 2016). Microorganisms can also directly promote plant growth by providing or regulating the levels of essential plant hormones (Zhang et al., 2008), such as growth hormone, cytokinin, ethylene, and gibberellin. Many Gram-positive and Gram-negative bacteria, including *Bacillus* spp., and *Streptomyces* spp., have been reported to produce indole acetic acid (IAA), cytokinins, gibberellins, and abscisic acid (Raddadi et al., 2008; Ali et al., 2017). In addition, microorganisms can also indirectly promote plant growth by reducing the inhibitory effect of various pathogens on plant growth (Perez-Montano et al., 2014; Sanchez-Lopez et al., 2016; Chaurasia et al., 2018). NL-11 and NL-1 used in this study belong to *Bacillus* and *Streptomyces* spp., respectively, which are gram-positive organisms. They not only promote plant growth by releasing mineral nutrients through solubilization, but also have been reported to produce IAA in our previous experiments, which is consistent with the report of Raddadi et al. Furthermore, this result enriches the pool of IAA-producing strains.

We used entropy-AHP to calculate and rank the weights of each strategy. The APG method was developed by combining the findings of various previous studies on water retention agents and substrates. To verify the practical application of the APG method, we selected a rocky slope of a quarry in Xiashu Town, Zhenjiang, as a test site for revegetation using the spray seeding method, and the substrate was prepared using a soil mixture (specific configuration:15 g/m^2^ of seeds, 5 kg/m^2^ of guest soil, 10 g/m^2^ of wood fiber, 40 g/m^2^ of organic fertilizer, and 100 g/m^2^ of peat soil) inoculated with MSMs. As a blank control, no MSMs were applied in one strategy. After 6 months of construction, the regreening effect of the APG method was observed to be clearly superior to that of the control (**Table 5**).

**Table 5:** APG method slope greening effect.

Index			A	B	C	AB	CK
**Plant growth**	**Shrubs** **(*Amorpha fruticosa* Linn.)**	**Survival rate (%)**	92	80	71	86	63
		**Height (cm)**	53.8	45.2	40.7	47.9	37.5
		**Diameter (cm)**	0.71	0.55	0.48	0.62	0.34
		**Crown size (cm)**	33.2	25.3	28.5	31.4	25.1
	**Herbal** **(*Lolium perenne* Linn.)**	**Emergence rate (%)**	54	41	43	50	39
		**Survival rate (%)**	52	30	36	47	28
		**Height (cm)**	22.7	18.5	18.4	20.2	15.3
		**Coverage (%)**	30	25	25	26	22
**Soil condition**		**Soil erosion**	+++	++	++	+++	+
		**Soil stripping**	***	**	**	***	*

+: Obvious gouging; ++: A small amount of loss; +++: Essentially no churn. *: Severe peeling; **: Slight peeling; ***: Essentially no peeling.

## 5 Conclusion

The scientific protection and construction of slopes are of great importance for social economic and environmental security. Accordingly, the research and development and promotion of slope management technologies are being strengthened worldwide. This study presents a preliminary exploration of the APG method based on patented MSMs. The results showed that MSMs can significantly increase the content of metal ions in the soil, increase nutrient availability to plants, improve soil fertility, and create an ideal environment for the growth of plants on slopes. At the same time, MSMs can also strengthen the growth of plants and roots, improve nutrient supply, which is conducive to improve the adaptability of plants in the process of slope restoration. An entropy-AHP analysis of the weights of different strategies on plant, root system, soil, and integrated dimensions revealed that the strategy with NL-11 added had the most superior integrated performance.

MSMs can promote the weathering of rock wall minerals and accelerate the degradation of rocks, improve soil conditions, promote the penetration of plants and roots, and maintain the stability of slopes. Therefore, they have great potential in slope regreening. Considering the results of various previous studies on water retention agents and substrates, the APG method based on MSMs was applied to field trials, and the results showed that the APG method provides clearly better effects than the traditional greening method.

The APG method has great potential for application in slope management and is worthy of further in-depth study and promotion. In order to realize the large-scale application of this method in slope management and scientific greening, there is still a lot of work to be done, such as the specific configuration of the soil mixture; the preparation, preservation and transportation of MSMs; the coordination of ecological rationality and economic feasibility; and the study of the geographical adaptability of APG, etc.

## 6 Patents

Jinchi Zhang, Guanglin Wang, Bo Zhang, Yanwen Wu: An efficient limestone erosion bacterium *Bacillus thuringiensis* NL-11 and its application. CN103087954B; Jinchi Zhang, Guanglin Wang, Li Wang, Bo Zhang: An efficient limestone erosion actinomycetes *Streptomyces thermocarboxydus* NL-1 and its application. CN103103151B; Guanglin Wang, Jinchi Zhang, Jie Lin, Rong Cao: An efficient limestone erosion fungus *Gongronella butleri* NL-15 and its application. CN103087926B.

## Data availability statement

The original contributions presented in the study are included in the article/supplementary material. Further inquiries can be directed to the corresponding author.

## Author contributions

LW: Conceptualization, methodology, software, validation, visualization, investigation, data curation, writing- original draft preparation, funding acquisition. XT: Methodology, software, validation, visualization, investigation, data curation. XL: Conceptualization, methodology, software. JZ: Conceptualization, methodology, visualization, funding acquisition.
